# American Dental Association

**Published:** 1897-02

**Authors:** 


### Reports of Society Meetings.

### AMERICAN DENTAL ASSOCIATION.

(Continued from page 29.)

*August 6, 1896.—Third Day.—Morning Session.*

THE meeting was called to order at 9.30 A.M. by the President, Dr. J. Y. Crawford.

The minutes of the previous session were read and approved.

*Dr. Harlan.*—According to a rule adopted at Old Point Comfort two years ago, the two divisions of the Executive Committee were to provide for two general addresses to be delivered to the American Dental Association on the second and third day. This year I notice that that is omitted from the programme, and that the minutes of the last year's meeting do not show that any persons were appointed to deliver those addresses. If the Executive Committee do not desire to provide for that, I think it would be a good plan to have the gentlemen who are to deliver the addresses nominated from the floor and confirmed by the Association, and so take it out of the hands of the committee. It would have added very much to the success of the meeting if the addresses had been provided for this year. It would be a good plan, and would take away something of the narrowness of the addresses that are made from the sections, as they would be on some topic of general interest. If the Executive Committee would provide for these addresses, I should be satisfied; but if they do not, I think the members should take the matter into their own hands.

*Dr. Crouse.*—The Executive Committee owe the Association an apology. We had forgotten the matter, as it was a new movement, and the matter was not thoroughly carried out last year.

I made a partial report on behalf of a committee appointed to promote or increase the interest between the local societies which make up this body and this body itself. The duties of that committee were very imperfectly performed last year. The chairman of the committee has gone away, and requested me to say that he must be relieved from his duties. I think the work that is designed to be performed is probably as important as that of any committee of this Association. I move that we have a continuation of the work, to be performed by a committee appointed by the chair. It is a mistake to expect members of the profession to come here and, receiving no benefit, to continue to do so from year to year. This Association will be less than an ordinary mass-meeting, unless we get the co-operation of the local societies and their delegates in the way of making reports in the sections. I shall make another effort in that direction if the chair sees fit to appoint me on that committee.

*Dr. Fillebrown.*—I second that motion. I am heartily in favor of this proposition of Dr. Crouse's. It looks to me exceedingly practical, and a line on which we will make the society interesting. I would like to see the local societies interested, so that they shall examine the papers that are presented to them during the year, select their premium paper, have it put in proper shape, with drawings properly elaborated, and bring it here to have it read by the delegate, so that the delegate may come fully accredited with the reading of the paper that has been read in his local society. Then the transactions of this society will represent the scientific and literary production of the year. I want to see so many papers come up that this society can have its selection, that it will be considered an honor to have a paper read here, and instead of accepting anything that comes, without reference to its quality or quantity, we shall have those papers put under a censorship. We will then have the character of our proceedings immensely improved. The present rule of the society is that any paper that has been read by title in the local societies cannot be presented here; but a man will not wait a whole year to say here what he has to say. He will give it to his own local society.

Dr. Fillebrown reported on behalf of the Committee on the Union of the American Dental Association and the Southern Dental Association. He said that at the meeting of the Southern Dental Association after the appointment of this committee (as it was not held until after the last annual meeting of this Association), it was impossible to have any correspondence on the subject or to take any action in the matter during the years 1894 and 1895. At a meeting of the Southern Dental Association, held in Atlanta in October, 1895, a committee was appointed to respond to the invitation of the American Dental Association. The committees have had considerable correspondence during the year, and report the following communication from the Southern Dental Association:

"That the committees have been unable to come together, and, as a result of the correspondence, advise that an effort be made to have both societies hold their next meeting at some time and place which the committees might agree upon, and the Associations dispose of the matter. The Association recommended the matter to the committee to make final report to this society at its next annual meeting, and state that it would meet the approval of the Southern Dental Association if the American Dental Association will meet at Old Point Comfort on the first Tuesday in August, 1897,—the same time and place that the Southern Dental Association has voted to meet.

"S. W. Foster,

"*Secretary,*

"*For the Committee.*"

Your committee recommend the acceptance of the suggestion of the Southern Dental Association as to place of meeting, and also that the committee of the American Dental Association be continued another year.

A motion was made to adopt so much of the report as does not conflict with the by-laws of this Association and the programme adopted for this meeting.

Motion carried.

Dr. Barrett, as chairman of the Committee on the Revision of the Constitution and By-Laws, reported as follows:

We have made our report years ago, and it has been from time to time put off until I supposed the subject was entirely forgotten. At that time there were some provisions adopted by the committee after a great deal of thought, wide correspondence, and careful consideration of the whole subject, and it seemed to the committee that it would be wise for this body to adopt them. I think what I thought then,—that it should be considered, and it would facilitate the objects of this meeting very much.

*Dr. Holly-Smith.*—I move that the Association accept the report, and that the committee be continued.

*Dr. Crouse.*—We thought we had a good reason for deferring action on that report, on account of the extreme anxiety that those who have thought of the subject carefully have felt, of having one grand association that should be national in its character, and we did not wish to change our by-laws until the question was settled. If we do, of course those coming to unite with us (or we with them) will want to have something to say as to what the constitution should be. The society has not ignored the report, but it has been postponed to get the union of the profession in one national organization.

*Dr. Holly-Smith.*—I made the motion to continue because I thought the committee was in a position to be referred to by any future organization of this society, as part of another organization, and I thought it would be well to keep that committee intact until we might come to them and ask them to help us in another work. I would amend the motion by asking that the committee be continued and the report received.

*Dr. Barrett.*—This committee has never felt offended by the action of the Association, but we thought the subject did not meet with approval and that the time was not ripe for the reception of the work of the committee. We are willing to do whatever we can to forward the best interests of the Association.

Motion carried.

A motion was made to pass Section VII., Anatomy, Pathology, and Surgery.

Motion carried.

Section I.—Prosthetic Dentistry, Chemistry, and Metallurgy—was then called. Dr. Boice stated that the chairman of the section had been unexpectedly called away. The section has nothing to present, and they regret it very much, as they recognize the importance of the work of that section.

Section II., Dental Education, Literature, and Nomenclature.

*Dr. Ottofy.*—Last year Section II. took up so much time that this year it intended to make a very brief report. Had we known that all the sections would decline, we would have made a much longer report.

Dr. Ottofy then read the report of his section, of which an abstract follows:

Since 1887 this section has regularly reported the number of dental colleges in active operation in the United States, with such additional information as has been deemed proper. The rule followed simply aimed at a truthful report of the number of colleges, newly-organized institutions, and such other incorporated bodies as conferred the dental degree. No attempt to discriminate has been made. Inasmuch as the list thus published by this Association each year is being accepted and offered as evidence of the existence of dental institutions, it seems manifestly improper that institutions reputed to exist, but regarding which no information can be obtained, should be placed side by side with institutions which are established and known to be engaged in the work of dental education.

The innovation, therefore, begins this year of separating the dental schools of this country according to the information in possession of this section, dividing them into three classes, to wit:

1. Dental colleges in active operation.
2. Dental colleges organized during the past year.
3. Corporations conferring the dental degree, but regarding which no further information is at hand.

There is a total of forty-six dental colleges in active operation, three of which were organized during the past year, and three corporations conferring the dental degree.

There were matriculated as students of dentistry during the past year six thousand two hundred and ninety-three persons, six thousand and thirteen of whom were in actual attendance, and upon fourteen hundred and forty-six of whom the dental degree was conferred.

It is deserving of mention that every college now actively engaged in teaching, or which has been organized during the year (except one whose announcement has not yet been published), is either a member of the National Association of Dental Faculties, or an application is pending, or it has announced its intention to conform to the rules of the National Association of Dental Faculties. In the latter class there are but three colleges in the country, and these intend to apply for membership as soon as they have complied with certain necessary requirements.

This information might not be considered of much consequence, were it not for the fact that the National Association of Dental Faculties has, at its session this week, taken one of the most important steps in the history of dental education, by the adoption of a plan for the admission of students to colleges. Hitherto an applicant for admission presented himself for admission, and it was left wholly to the judgment of some official of the college to accept or reject. There is no doubt that this imperfect system resulted in the admission of many who were not properly qualified. By the plan just adopted, the requirements for admission into any of the colleges are identical, and the requirements are graded from year to year in such a manner that, beginning with next year, the educational standing of our students will be higher and increase within four years to a plane practically equivalent to a high-school education. The section deems this action one of the most important advances made in dental education.

The section has no further report to make on dental education or literature, desiring these subjects passed without discussion, in view of the amount of time devoted to them last year.

Dr. L. P. Bethel, of Kent, Ohio, then read a paper, entitled "A Suggestion regarding a Possible Means for instructing the Public in Dental Matters." An abstract follows:

If it be desirable to educate the public in a popular way regarding oral and dental hygiene, etc., some active steps should be taken, and this representative body—the American Dental Association—is the one to take the matter in hand. The subject has been discussed in our societies time and again, but this has not benefited the public, for we have taken no active steps towards instructing them. The following plan seems to the writer to be the most feasible of any yet presented:

Let the American Dental Association appoint a committee.

The duties of this committee shall be, first, to correspond and arrange with leading dailies in the United States, one only in each city, to publish one prepared article in the Sunday edition each week until a whole series is printed, the articles to go to the publishers thoroughly edited and ready for publication, and to be gratuitously given, provided an announcement be made each week in the daily, advertising its Sunday edition, calling attention to the forthcoming article. The Sunday paper is suggested because it has a larger circulation and is more generally and thoroughly read than the dailies, although the Saturday or Monday issue could be used if desired. If this be accomplished, the next duty of the committee shall be to appoint prominent men in the profession to write a series of articles in popular style, bearing upon every desirable phase of the subject in hand, each person to write one article only, and that to contain not more than one thousand to fifteen hundred words. These articles, when written, shall be sent to the committee to be edited for the press. When set in type, proof will be sent to the author for revision. When corrected, enough copies will be printed from the galley to supply the papers secured, proofs of each article to be sent publishers about one week in advance, and the article to appear simultaneously in the desired edition of all the papers, and this continued each week until the whole series is published. There are about fifty available papers, and they reach fully twelve million readers. Then, if desired to further extend this knowledge, dentists in towns can get their own paper to republish the articles without credit and call especial attention to them. When set in type, the matter can be paged and stereotyped for pamphlet use, if desired. The pamphlets would be very cheap, and enable dentists to supply their patients and also teachers in the public schools, that they may read portions of the articles to pupils in the school-room. The expense of carrying out this plan would include type-setting, stereotyping, and postage, and reasonable compensation should be given to a member of the committee who attends to the proof-reading, stereotyping, etc., for the time spent by him. The whole expense would not be great. If each State and local society would contribute a few dollars, aside from that given by the American Dental Association, there would be ample funds to carry this into effect, and who could estimate the benefit to millions of people?

*Dr. Ambler.*—What I say will be in reference to the last paper which was read. I have always been a firm believer in the fact that the more we educate the public the better it will be for them and the better it will be for the dentist himself. It makes a practical thing of it and brings it right home. It will assist you in every-day practice. This idea of Dr. Bethel is a very good one indeed, and I consider it a practical thing. A great many, at first thought, would say it will not amount to anything, that every man will be advertising himself, and it should not be before the National Association. I feel otherwise. In 1872 the editor of a weekly paper called *The Ohio Farmer*, which had been published in Ohio since 1846 (and with whom I was acquainted), asked two or three dentists in the city of Cleveland to write a few articles for the popular education of the public. He did not succeed, and finally he came to me and asked me to do it. At first I declined, thinking it would be considered an advertisement; but after a second thought and further talk in regard to the matter, I concluded to do so. In *The Ohio Farmer* of 1872 you will find a series of articles published weekly, entitled "The Care and Preservation of the Teeth," written in a plain, popular style, so that any one could understand them. So you see I am in hearty accord with this idea of Dr. Bethel's. I presume there are other gentlemen here who have done the same thing in times past. I believe it to be valuable, and if we could put it into practical operation I would vote for it.

*Dr. Templeton*, of Pittsburg.—I have a few words to say on this subject. What I shall say is in reference to the last paper. I endorse it all except one thing; I believe in the Christian religion, and I am opposed to the Sunday portion of the report. It is not necessary that we should support Sunday newspapers. I do not buy the Sunday newspapers when I am at home, and I cannot vote to put anything in a Sunday newspaper.

*Dr. Wilson.*—I would like to ask Dr. Templeton if he buys the Monday morning paper. If so, which does he patronize,—the Sunday work or the Saturday work?

*Dr. Molyneaux.*—Is this not a good Christian cause right along? I think the Sunday paper is a very good paper to put it in.

*Dr. Taft.*—The committee contemplated in the paper of Dr. Bethel would be a permanent committee to carry out the recommendation, not to consider whether the question should be adopted. I think the proper course would be to move the adoption of the suggestions in Dr. Bethel's paper, and I make such motion.

Motion seconded.

*Dr. Barrett.*—I am opposed to anything of that kind. We want to keep out of the newspapers. If we put articles into the newspapers, we follow the precedent that has been set by the disreputable portion of the profession. It is very nice and pretty to talk about the education of the people through the newspapers, but for one insertion that we will make, ten will be made by the other side, of propositions that we do not desire to sustain. We cannot place it so emphatically before the people but that the quacks, who have more experience in this advertising matter and have personal ends to gain, will emphasize their own standing ten times more. The eagle is a very ungainly bird when he attempts to walk on the earth. The barn-yard fowl is very graceful when he crows and struts, but the eagle can soar into the empyrean, while the other is of the earth, earthy; and this advertising, whether it comes from the profession or from the people who oppose the profession, is of the earth, earthy, and you cannot elevate it into the upper strata by any means that you seize upon to make it respectable. You are adopting the methods of the enemy, and they are naturally debasing in their character. We cannot afford to have anything of that kind. If the American Dental Association adopts this, I cannot endorse it.

*Dr. Morgan.*—Dr. Barrett is quite correct on this subject. I do not think that the gentlemen of this Association, if they consider carefully what these suggestions involve, would be willing to undertake them, because of the immensity of the thing, in the first place. In the second place, if you adopt that plan, you invite the whole rabble of cheap dentists into the papers in competition with you. They will put in two or three articles to your one, and they will mislead the people more than you will educate them and correct their views. You simply invite all that class of men that this Association has ignored and debarred from the privileges of the Association to meet you on ground where they have the advantage of you very largely. Let them alone. Keep out of the newspapers. There are other means that we can adopt for the education of the people. Very few people accept what is published in the newspapers, and if it is published in the interest of any particular class of individuals, whether professional or not, it at once comes under the ban of suspicion, unless you will put your name to it, which I do not think you will care to do. If you want to educate people by publications, make them of your own, where you have entire control of them, and where you can put them where you desire and keep within the bounds of professional dignity. Do not invite the sort of thing that we would get from the publication of these things in the secular papers.

*Dr. Barrett.*—I move that so much of the report as refers to publications in the newspapers be laid on the table.

Motion carried.

A motion was made to receive Dr. Ottofy's report and place it on file.

Motion carried.

Dr. Guilford then reported on behalf of the Special Committee on Nomenclature as follows:

Your committee would report that it has endeavored to carry forward its appointed work to the best of its ability. The tabular portion of last year's report, which could not be read on account of its length, has since been carefully revised, and appears in the published transactions. Notwithstanding the great amount of time spent on it, it is not entirely free from errors and inaccuracies. Some of these have been called to the attention of the committee, and it is hoped that others will be. While the report was in type, several hundred reprints were made and sent to individuals in different parts of the country, with the request that the report be brought before the societies. This was done in several cases, but the results have been published in one instance only,—namely, the Academy of Stomatology of Philadelphia. Its consideration of the subject is printed in full in the August number of the INTERNATIONAL DENTAL JOURNAL, and parts of it are incorporated in the present report.

Your committee had some difficulty in deciding on a system of phonetic spelling in indicating the sounds of vowels and syllables, but finally decided to adopt that used in the "Century Dictionary."

Your committee has thought best to append a list of terms the use of which should be discontinued, and a brief summary of the discussion at the Academy of Stomatology of Philadelphia.

We have also a short paper from Dr. Thompson, a member of the committee. He says it was the late lamented Dr. Kulp who said that the nomenclature of a profession is the sign-board of its intelligence and training, or, as Dr. Ward has said, we can no more dispense with nomenclature than we can with language. It is the language of science, and as such should possess all the precision that science requires in all departments. Those who regard it as of no value should not forget that the great Darwin considered the subject of nomenclature of such paramount importance that he actually bequeathed a sum of money to be devoted to it. All scientific workers, no matter what branch of science they pursue, feel the same,—that the language of science and the nomenclature of its facts, especially in the organic world, should be reduced to the most perfect form for their use.

I would state in connection with this that Dr. Thompson stated, in a letter he sent to me, that the work of the committee had received the approval of one of our highest scientific authorities in America,—Dr. Cope, of Philadelphia.

*Dr. Barrett.*—I can most heartily commend this report, and yet I am inclined to think it oversteps the bounds that should be prescribed for the work of this committee. It is not a committee on philology. It has for its especial sphere the consideration of dental nomenclature, and if we go outside of that into the general field of philology, we are opening something that will be so broad and so wide that it will take the place of other matter which we want to discuss. The sole criticism upon the paper is that it goes beyond the bounds which should be prescribed for the consideration of the subject before the committee, and enters into the general field of philology, which does not belong to us to consider. With that single criticism I most heartily commend the paper, because it is in the line of general advancement.

*Dr. Taft.*—I should like to know how we are going to study the subject of nomenclature without words. Dr. Barrett condemns the report in that particular,—that it enters upon the consideration of the subject of philology. If he can separate philology and nomenclature, he can do what some of us cannot do.

*Dr. Barrett.*—We are supposed to have some knowledge of general literature and of the general nomenclature. The subject for consideration of this body is that which is professional. When we enter the general field of philology, it is too wide for our consideration, as I said before.

*Dr. Stellwagen.*—Having done a little work in that direction, I can appreciate what the labor of this committee has been, and while I heartily commend it, I think the committee will probably feel more complimented if, while accepting its report, we put off the acceptance or endorsement of these words until we have seen them in print and examined them. Therefore I think next year would be time enough to endorse the pronunciation. Some of the words as they were read seemed a little peculiar, and I think it would bear a close examination into the etymology and meaning of the various forms through which these words have reached the English language. It would be profitable to us all to study them. I hope in accepting the report we will not accept the conclusions as to pronunciation.

*Dr. Guilford.*—The committee that has undertaken this work has pursued it with the sole object of the good of the profession. We want to do what we can in the way of dental education, and we are glad to receive criticisms or suggestions. If the gentlemen have any objections or criticisms to make, we will be very glad to receive them. The work can be greatly improved upon, but we cannot improve it without your help.

Motion carried.

Dr. Watkins stated that Section III., Operative Dentistry, had nothing to offer. Dr. Fuller did not know that he was elected chairman of the section until a few days before this meeting.

*Dr. Crouse.*—We have adopted a programme for this meeting, which sets the hour of eleven o'clock to-morrow for selecting our next place of meeting, election of officers, and other miscellaneous business. I think it would be well to rearrange our programme and have the meeting this evening at five o'clock. I would make a motion to that effect. Many gentlemen have requested me to make this motion so we could get through with the business of the Association.

Motion carried.

The chair appointed on the Committee on National Museum the following gentlemen: Drs. Donnelly, Taft, McKellops, Henry W. Morgan, and Fillebrown.

Drs. J. N. Crouse, L. P. Bethel, and A. W. Harlan were appointed on the Committee to promote Interest in State and Local Societies.

*Dr. Crouse.*—This committee wants power to make its own selection in the local societies as to who shall make the digest of the work done during the year.

*The President.*—As there is no objection, your committee will have that power.

Dr. Henry W. Morgan was substituted in place of Dr. Rhein, resigned, as a member of the Committee on Nomenclature.

The Executive Committee nominated the following gentlemen to address the Association at the meeting in 1897:

Dr. A. W. Harlan; subject, "Miasmus and Infection."

Dr. Cassidy; subject, "The Relation of Chemistry to Dentistry."

*Dr. Taft.*—The Committee on Necrology has found it impracticable to meet, as some of the members have gone, and it has not been found possible to formulate the notices during this meeting. At the suggestion of Dr. Pierce, we ask that these notices be prepared within a few days and sent to the secretary.

The request of the committee was granted.

*Dr. Carroll.*—I was advised by the chairman of Section VI. that he had not been able to get his committee together, and hence whatever matter there might be before the section had not been presented to this body. I prepared a paper at the request of the chairman of that section, and I learned this morning that he was not able to get his committee together, and hence that paper would not be presented; therefore I ask that it be returned to me. I have been a member of the society since its organization, and I have never before prepared a paper. This time I did, and the paper is returned without the section being called together.

*The President.*—The chair would state that it is only competent to have before this body such matter as may come through the regular channels of the sections, either reported upon by the chairman of the section or the secretary. The secretary of Section VI. stated that he had reported all the matter that had been presented to him.

Dr. Corydon-Palmer was accorded time in which to present what he wished to say to the society. He spoke as follows:

Yesterday I unexpectedly said part of what I expected to say to-day. As I referred to the past a great deal, I will not say much about it; but I wish to make these statements, that I am not in trade, and what I have done has been in the way of artistic design. I have given everything I have to the profession and to this Association. I do not wish to be rated as an instrument-maker. My desire has always been to be known as a fine operator. If, in my daily practice and that which I consider to be art in operating, I conceived the idea of certain appliances or adaptations, I have been happily able to produce them myself, without putting them into other hands to be brought forward. What I have to-day I prepared especially for this meeting and as a continuation of what I did in 1873. It is a revision of the instruments for putting holes in the rubber dam. At that time a large case of instruments was offered, and it contains instruments in the form which would place the hole in the rubber dam and enable one to extend it when putting it on over other teeth. I have encountered opposition in the making and offering of my appliances because people said the principle was false, that the steel would cut, and that I was all wrong. Notwithstanding, I have been able to do it myself and have had it in use ever since. These are my gems, and I wish to preserve them carefully. I brought forward the idea of having a curve in the joint of the instrument, so that in using it in and out the joint is out of the way, which enables one to see what is being done. Here is a small-sized one for putting in wedges between teeth and taking them out. That is its principal use, but it is also useful to take off ligatures. You all use wood for wedges. The orange-wood is not better than straw. Here let me say with all honor and appreciation of the separators which we have,—the Perry and the Bonwill and others,—that they all have their places; but notwithstanding that, we sometimes need other wedges. This little instrument is the one to put them in with and take them out, and you can use it in the back part of the mouth and look in and see what you are doing. For wedges you should use nothing but the finest quality of Turkish boxwood. Cut it in suitable strips, and you have something you can rely on.

Here is my wedge-cutter. It is curved. Mr. White, who favored all of my appliances, opposed me in this. He said the joint was false and was not in accordance with true mechanical principles; but I said if the surgery of the mouth calls for it, it should be made in this way. You can reach any part of the mouth with this and see what you are doing. The cutting part is not so broad as to be liable to clip a hole in the dam, which the other cutters do. If these instruments are reproduced, and you get one, do not use it for anything except wood or upon a hard substance, because you will spoil it if you do. Do not use it for the engine bits; there are pliers made for that purpose. This is for wood only.

Here I have three sizes of instruments for cutting holes in the rubber dam. They are numbered, and make three different-sized holes. They cut upon steel and cut the piece out nicely. If you put the dam in the mouth and you wish to extend it over another tooth, which is very frequently the case, this will reach any part of the mouth, and you can put the hole wherever you wish. I have made the instruments entirely myself. It is the work of my own hands. There is an advantage in what I am doing, because, knowing the use of the instruments, and the want of them, I have made them just as they were needed.

A vote of thanks was offered to Dr. Palmer for his kindness in exhibiting these instruments.

*Dr. Stellwagen.*—I would like to say that I have had one regret in my lifetime, and that was that once, in speaking of these instruments or some similar ones which Dr. Palmer had called my attention to, I said they were poetry in steel. After I had put the manuscript into the hands of a dear friend for revision, he objected to the phrase, and I allowed that language to be changed. I have thought ever since that I was cowardly because I had permitted it to be changed. I repeat now that those instruments are poetry in steel. I have used instruments similar to them for years, and for sweetness of cutting those wedge-cutters seem to me always as if I were cutting through oil rather than through wood.

Adjournment.

(To be continued.)

